# The Combination of Phytogenic in High-Starch Diets for Growing Cattle and Its Effects on Immunomodulation, Fermentation, Microbiota, and Performance

**DOI:** 10.3390/ani16172803

**Published:** 2026-09-06

**Authors:** Alexandro Fritzen, Maisa Damo, Ana Lara Amaral da Veiga, Jean Carlos Martinotto Piaceski, Letícia Lescano Neves, Renato Santos de Jesus, Suelyn de Oliveira Marques, Miklos Maximiliano Bajay, Luiz Eduardo Lobo e Silva, Roger Wagner, Gilnei Bruno da Silva, Daiane Manica, Margarete Dulce Bagatini, Aleksandro Schafer da Silva

**Affiliations:** 1 Multicenter Postgraduate Program in Biochemistry and Molecular Biology, Universidade do Estado de Santa Catarina, Lages 88520-000, SC, Brazil; fritzen.vet@gmail.com (A.F.); miklos.bajay@udesc.br (M.M.B.); gilnei.silva@edu.udesc.br (G.B.d.S.); 2 Department of Animal Science, Universidade do Estado de Santa Catarina, Chapecó 89815-630, SC, Brazil; maisa.d@edu.udesc.br (M.D.); ana.ladv@edu.udesc.br (A.L.A.d.V.); jean.cmp7@edu.udesc.br (J.C.M.P.); leticia.neves21@edu.udesc.br (L.L.N.); renato.jesus0026@edu.udesc.br (R.S.d.J.); 3 Postgraduate Program in Animal Science, Universidade do Estado de Santa Catarina, Chapecó 89815-630, SC, Brazil; suelyn.marques@edu.udesc.br; 4 Department Food Science and Technology, Universidade Federal da Santa Maria, Santa Maria 97105-900, RS, Brazil; eduardo.lobo@acad.ufsm.br (L.E.L.e.S.); rogerwag@gmail.com (R.W.); 5 Graduate Program in Biochemistry, Universidade Federal Santa Catarina, Florianopolis 88040-900, SC, Brazil; daianemanica2011@gmail.com; 6 Department of Biochemistry, Universidade Fronteira Sul, Chapecó 89815-000, SC, Brazil; margarete.bagatini@uffs.edu.br

**Keywords:** microbiota, gut health, performance, feed efficiency

## Abstract

High-starch diets can impair gastrointestinal homeostasis in growing cattle by promoting dysbiosis, inflammation, and oxidative stress. This study evaluated the effects of a multi-active phytogenic blend containing *Macleaya cordata* extract, essential oils, live yeast, silymarin, bentonite, and beta-glucans on performance, inflammatory and oxidative biomarkers, volatile fatty acids (VFA) production, and microbiota composition in calves fed a high-starch diet. Twenty-eight 5-month-old Holstein males were assigned to a control diet (control group) or the same diet with addition of 15 g/animal/day of the additive (Phytogenic group) for 70 days. Calves fed with additives had greater weight gain and average daily gain (*p* = 0.05), whereas dry matter intake and feed efficiency were unchanged. The additive reduced total leukocytes and lymphocytes number, decreased of biomarker in serum as TNF-α, IL-1, C-reactive protein, ferritin, haptoglobin, creatine kinase, cholinesterase, TBARS, and reactive oxygen species. These animals of the Phytogenic group also have increased IL-10, IgA, IgG, total protein, and globulin concentrations compared to the control group. Ruminal and fecal VFA concentrations were higher in animals of Phytogenic group, including acetate, propionate, and butyrate. In addition, intake of additives increased ruminal microbial richness and altered beta diversity, indicating a marked shift in rumen microbial structure, while altering the relative abundance of specific taxa associated with high-starch feeding. These findings indicate that this phytogenic blend improves growth performance and exerts immunomodulatory, antioxidant, and microbiota-modulating effects, supporting its use as a nutritional strategy to mitigate the metabolic challenges associated with high-starch diets in growing cattle.

## Simple Summary

High-starch diets can impair gastrointestinal homeostasis and animal performance by promoting dysbiosis, inflammation, and oxidative stress. We evaluated a novel multi-active feed additive in Holstein calves fed a high-starch diet. Animals what receiving the additive exhibited greater weight gain, reduced inflammatory and oxidative biomarkers, enhanced immune responses, increased ruminal and fecal short-chain fatty acid concentrations, and improved rumen microbial diversity. Furthermore, the additive selectively reshaped the ruminal microbial communities. These results indicate that the additive effectively mitigates the negative impacts of high-starch feeding and represents a promising nutritional strategy to improve health and productivity in growing cattle.

## 1. Introduction

In cattle, the ability to utilize pastures is associated with the activity of their ruminal microbiota [1], which is established through colonization after birth, and its functionality affects the health, performance, and efficiency throughout the ruminant’s life [2,3]. Among the most common bacterial species, 67.1% belong to the genera *Prevotella, Butyrivibrio, Ruminococcus,* and unclassified Lachnospiraceae, Ruminococcaceae, Bacteroidales, and Clostridiales [1], with diet having a marked effect on the diversity and composition of the microbiota [4]. In diets with high starch levels (≥27.7% of dry matter), hindgut dysbiosis was observed, demonstrating the modulating effect of diet on the gastrointestinal microbiota [5].

Diets with a higher inclusion of roughage and fiber increase cellulolytic bacteria and lead to an increase in ruminal pH with increased acetate production by bacteria of the genera *Ruminococcus, Fibrobacter*, and *Butyrivibrio* [6]. It has been observed that with the increase in concentrates and starch, there is an increase in the genera *Prevotella, Ruminobacter, Selenomonas*, and *Succinomonas*, raising propionate production [2,6], with an increased risk of subclinical ruminal acidosis and elevated levels of lipopolysaccharides derived from Gram-negative bacteria, associated with increased expression of inflammatory cytokines and levels of reactive oxygen species [7,8].

The interaction between the immune system and the gastrointestinal microbiota is fundamental for the maturation of the immune system after birth and for the development of balanced responses to bacteria and other microorganisms [3]. The development and maintenance of gastrointestinal health is promoted by metabolites produced by fermentation, with an increase in butyrate helping to improve the health and integrity of cellular barriers [9]. Substances such as beta-glucans and mannan-oligosaccharides act in the development of an efficient immune response and competitive exclusion by binding to pathogens, respectively, demonstrating the ability of diet to modulate the microbiota and the immune system [10].

The use of phytoactives and essential oils shows great potential in modulating the microbiota and volatile fatty acid profile, modulating the immune response, and reducing oxidative stress, thus increasing efficiency and production [11,12]. However, the combination of substances has varied effects with additive, synergistic, and antagonistic interactions, highlighting the need to evaluate the composition of blends and the doses of their active ingredients to establish efficacy [13]. Phytoactives derived from *Macleaya cordata*, which are rich in quaternary benzophenanthridine alkaloids such as sanguinarine, have demonstrated strong anti-inflammatory and antimicrobial properties, effectively modulated nutrient degradability, and reduced systemic inflammation in ruminants [14]. Concurrently, the inclusion of live yeasts (*Saccharomyces cerevisiae*) serves as a well-established strategy to stabilize ruminal pH under dietary challenges; and live yeasts actively consume oxygen within the rumen, creating a strictly anaerobic environment that stimulates the growth of lactic acid-utilizing bacteria and cellulolytic species, thereby minimizing the risk of subacute ruminal acidosis and enhancing fiber digestion [15]. However, the effect of the combination of active ingredients and its ability to mitigate the effects of high-starch diets are not well understood.

Maintaining gastrointestinal health and the balance of the microbiota affects hepatic and systemic health [16]. Studies show that diets high in starch increase the supply of LPS to the portal system, raising the hepatic challenge [17]. Bentonite has demonstrated the ability to absorb LPS, reducing its intestinal absorption [18], while silymarin can reduce inflammation and stimulate hepatoprotection by increasing hepatocyte cell regeneration [19].

We hypothesize that combining *Macleaya cordata,* essential oils, live yeasts, silymarin, bentonite, and beta-glucans synergistically mitigates hindgut dysbiosis and inflammatory challenge in growing cattle fed high-starch diets, thereby improving feed efficiency, immune response, and reduces oxidative stress. To evaluate this hypothesis, the present experiment assesses the effects of this multi-active blend in growing cattle fed a high-starch diet, analyzing performance, hematological, immunological, oxidative, microbiome, and metabolic parameters.

## 2. Materials and Methods

### 2.1. Product

For the development of this experiment, the product LactoPremium^®^ (a.z. Nutri, Toledo, Brazil) was used. This product was formulated based on a combination of other commercial products: Macleaya cordata extract, (Sangrovit^®^ 1.0 g, Phytobiotics Brasil, Cambé, Brazil), Carvacrol, eugenol, cinnamaldehyde, and red pepper resin, representing 450 mg of active ingredients (Activo-O.E^®^. 3,0 g, Grasp, Curitiba, Brazil), Live Yeast 1.5 × 10^10^ Colony Forming Units (CFU) (Procreatin^®^ 6.0 g, Phileo by Lesaffre, Campinas, São Paulo, Brazil) and combination of bentonite, Beta-glucan, silymarin, and selenium (Yes Fix HP^®^, Yes, São Paulo, Brazil). The composition and concentration of the product is shown in Table 1.

### 2.2. Facilities and Animals

The experiment was conducted at the Universidade do Estado de Santa Catarina Experimental Farm in the municipality of Guatambu, Southern Brazil, under confinement conditions. We used 28 intact male Holstein cattle, 5 months old, in this experiment. The animals were housed in pens (32 m^2^) of a partially covered barn, that is, 1/3 of the pen in the feeding area protected from rain and sun. Each pen had an individual feeder, as well as an automatic waterer, which assesses individual feed and water consumption. The cattle were allocated in pairs in the pen. Prior to the start of the experiment, it was verified that all animals were apparently healthy; in addition, all animals underwent a Pour-on antiparasitic protocol (Fluatac DUO**^®^**, Ourofino Saúde Animal, Cravinhos, Brazil).

### 2.3. Diet and Experimental Design

Using BrCorte 2016, we formulated the cattle diet in this experiment, in a proportion of 35% roughage (corn silage) and 65% concentrate (ground corn (26.7%), soybean meal (11.4%), wheat bran (15.9%); soybean hulls (9.58%), mineral/vitamin supplement (0.46%), common salt (0.30%) and calcitic limestone (0.45%)). The diet was planned for 14.1% crude protein and 71.5% TDN, within normal limits, however, exceeding starch levels (33.8%) to cause a nutritional challenge. The cattle were fed twice a day (08:00 h and 16:00 h) based on the formulation to meet nutritional requirements and maximize daily body weight gain by 1.25 kg. The 28 animals were then divided into two groups: Control (n = 14) and Phytogenic (n = 14), distributed in 14 pens (2 animal per pen). Knowing the daily concentrate consumption, the additive was added to this feed in a proportion that provided a dose of 15 g/animal/day. The experiment was conducted over 70 days, with dietary exposure and the adaptation period forming part of the evaluation model employed.

A completely randomized experimental design involving two groups was used in this study. When performance variables (body weight, weight gain, feed intake, and feed efficiency) were evaluated, the pen was considered the experimental unit; however, for the other variables (blood, ruminal fluid, and feces), the animal was the experimental unit.

### 2.4. Data and Sample Collection

Feed intake was measured daily by weighing the amount provided and, the following day, weighing the leftovers, which were discarded before the first feeding of the day. The animals were weighed on days 1, 14, 28, and 70 of the experiment using a digital scale. On the same weighing days, samples of the feed provided to the animals were collected, frozen (−20 °C), and used in a pool for chemical composition analysis. This data allowed the calculation of daily weight gain (DWG) and daily dry matter intake (DMI), information used to calculate feed efficiency (DWG/DMI).

On days 1, 14, 28, and 70 of the experiment, blood samples were also collected by coccygeal vein puncture. The blood was placed in tubes with EDTA anticoagulant to perform a complete blood count (up to 3 h after collection) and another part in tubes with clot retractor. The material was stored in a thermal box during collection and transport to the laboratory, at 2 °C to 8 °C in a thermal box. For serum collection, samples were centrifuged (750 g for 10 min), and the collected serum was placed in microtubes and frozen (−20 °C).

Rumen fluid samples were collected on day 70 only, using a silicone esophageal probe attached to a vacuum pump with the first 20 mL discarded to avoid contamination with saliva. The pH of the collected material was measured immediately (digital pH meter, Testo^®^, Campinas, Brazil); then a sample was collected using a swab for rumen microbiota, and the remaining material was filtered and frozen to measure VFA.

Feces were collected directly from the rectal ampulla using sterile gloves on days 1, 14, 28, 42, and 70 of the experiment to measure pH (digital pH meter, Testo^®^, Campinas, Brazil). Stool samples from day 70 were used in other analyses, namely, for intestinal microbiota (swabs) and VFA profile in stool (frozen material: −20 °C).

### 2.5. Laboratory Analysis

#### 2.5.1. Feed Analysis

Feed samples were dried and subsequently ground in a knife mill equipped with a 1.0-mm screen. Chemical analyses were performed to determine MS and PB according to the methodologies described by AOAC [20]. Ether extract (EE) concentration was determined using the lipid extraction method proposed by Bligh and Dyer [21]. Neutral detergent fiber (NDF) concentrations were determined according to the detergent system described by Van Soest et al. [22]. Starch content was determined using an enzymatic method as described by Hall [23]. Samples were subjected to hydrolysis with thermostable α-amylase and amyloglucosidase, followed by quantification of the released glucose by spectrophotometry. NDT was calculated based on nutrient digestibility coefficients according to NRC [24]. Results in Table 2.

#### 2.5.2. Hematological Analysis

Hematological variables were obtained using blood collection tubes containing EDTA and analyzed using the automatic hematology analyzer VET3000 (EQUIP^®^, São Paulo, Brazil), which determines leukocyte, lymphocyte, granulocyte, monocyte, erythrocyte, and platelet counts, as well as hemoglobin concentration and hematocrit.

#### 2.5.3. Serum Biochemistry

Serum concentrations of biochemical variables, including albumin, cholesterol, glucose, uric acid, and urea, were measured. Globulin levels were calculated mathematically (total protein-albumin). The activities of gamma-glutamyl transferase (GGT), aspartate aminotransferase (AST), creatine kinase, and cholinesterase were also determined using commercial kits from Analisa^®^ and an automated analyzer, Zybio EXC 200 (Shenzhen, China).

#### 2.5.4. LPS, Cytokines, and Proteinogram

Serum lipopolysaccharide (LPS) concentrations were determined using the chromogenic Limulus Amebocyte Lysate (LAL) method, with a specific commercial kit, Pyrochrome (Associates of Cape Cod Inc., East Falmouth, MA, USA), and results were expressed as EU/mL.

Serum concentrations of TNF-α, IL-1β, and IL-10 were measured using commercial kits from USCN Life Science Inc. (Wuhan, China) and an ELISA assay performed with the Chem Well (Awareness Technology Inc., Palm City, FL, USA) to determine cytokine concentrations in pg/mL.

Sodium dodecyl sulfate polyacrylamide gel electrophoresis was performed according to Fagliari et al. [25] using minigels (10 × 10 cm). Gels were stained with Coomassie blue and photographed for identification and quantification of protein fractions using the LabImage 1D software (Loccus Biotechnology, São Paulo, Brazil). A molecular weight standard containing fractions ranging from 10 to 250 kDa, Kaleidoscope (Bio-Rad Laboratories, Hercules, CA, USA), was used as reference.

#### 2.5.5. Oxidative Status

Lipid peroxidation in serum was measured by thiobarbituric acid reactive substances (TBARS), following Jentzsch et al. [26]. Serum reactive oxygen species (ROS) levels were analyzed by fluorescence according to LeBel et al. [27]. The activity of the antioxidant enzyme superoxide dismutase (SOD) in whole blood was evaluated according to McCord and Fridovich [28]. Serum protein sulfhydryl group (PSH) levels were determined spectrophotometrically using the reagent 5,5′-dithiobis-(2-nitrobenzoic acid) (DTNB), as described by Ellman [29].

#### 2.5.6. Volatile Fatty Acid Profile in Rumen Fluid and Feces

Rumen fluid and fecal samples were stored at −20 °C until volatile fatty acids (VFA) analysis. For analysis, samples were thawed at 5 °C and homogenized. Fecal samples (150 mg) were previously mixed with 750 μL of methanol. Subsequently, samples were centrifuged (12,300× *g* for 5 min), and aliquots of the supernatant were transferred to microtubes containing formic acid, followed by homogenization and a second centrifugation step. The supernatant was then added to a methanolic alcohol solution used as an internal standard, followed by homogenization and centrifugation. After preparation, 600 μL of the sample were transferred to injection vials.

VFA determination was performed by gas chromatography equipped with a flame ionization detector (GC-FID; Varian Star 3400/Chrompack CP-3800, Varian, Inc., Palo Alto, CA, USA) and an automatic sampler (Varian 8200CX/CP-8400, Varian, Inc., Palo Alto, CA, USA), using a CP-Wax 52CB capillary column (60 m × 0.25 mm × 0.25 μm), hydrogen as the carrier gas, and split injection mode (1:10). The analytes included acetic, propionic, butyric, isobutyric, valeric, and isovaleric acids (Table S1). Results were expressed as mmol L^−1^ of each VFA in the samples.

#### 2.5.7. Rumen and Intestinal Microbiota

On days 1 and 70, ruminal fluid and fecal samples were collected and preserved using 3M™ Quick Swabs for qualitative and quantitative detection of microorganisms through 16S rRNA gene sequencing. The analyses were performed at BPI—Biotechnology Research and Innovation^®^ (Botucatu, São Paulo, Brazil).

Total DNA was extracted from 200 mg (wet weight) of each sample using the ZR Fungal/Bacterial DNA MiniPrep Kit (Zymo Research, Tustin, CA, USA). The V3–V4 region of the bacterial 16S rRNA gene was amplified by polymerase chain reaction (PCR) with primers 341F (5′-CCTAYGGGRBGCASCAG-3′) and 806R (5′-GGACTACNNGGGTATCTAAT-3′). Library quantification was performed using quantitative PCR with the KAPA Library Quantification Kit (Illumina, San Diego, CA, USA), following the manufacturer’s instructions. Samples were then normalized to a final concentration of 2 nM and sequencing libraries were constructed and then sequenced on an Illumina MiSeq platform using 250 paired-end cycles, according to standard protocols.

### 2.6. Statistical Analysis

Data showed normal distribution after being subjected to the Shapiro–Wilk test; additionally, skewness, kurtosis, and homogeneity were evaluated using Levene’s test, while linearity was assessed using linear regression. Based on these preliminary results, data were analyzed using the SAS MIXED procedure (SAS Institute Inc., Cary, NC, USA; version 9.4) to determine the denominator degrees of freedom for testing fixed effects (day, treatment, and treatment × day interaction) in a completely randomized design to evaluate ADG and feed efficiency. Other variables (body weight, feed intake, serum biochemistry, oxidative status, and hematological parameters) were analyzed as repeated measures and tested for fixed effects of treatment, day, and treatment × day interaction (group), with animal considered as a random effect. All results obtained on d1 for each variable were also included as covariates, as well as initial body weight. Mean comparisons were performed using Student’s t-test. All results are presented as mean and standard error of the mean (SEM), with significance declared at *p* ≤ 0.05.

Sequence data were processed using Mothur v 1.48.3 [30], following the MiSeq SOP [31]. Taxonomic classification was performed by comparing representative sequences of each oligotype against the Greengenes2 database [32]. Closed-reference clustered operational taxonomic unit (OTU) tables were subsequently exported for community structure analyses in the R environment (v4.5.1; [33]), using the Phyloseq package (v1.52; [34]) and the Microeco package (v1.15.0; [35]). Potential contaminant sequences were identified and removed prior to downstream microbiome analyses using the decontam R package. The procedure was conducted separately for ruminal and fecal microbiota to account for the distinct microbial communities expected in each biological compartment. The decontam approach identifies putative contaminants based on statistical patterns characteristic of exogenous DNA, particularly increased contaminant frequency in samples with lower total DNA concentration together with taxonomic curation based on the expected microbiota of rumen and feces. A threshold of 0.5 was used for contaminant classification, with features showing a decontam score < 0.5 considered potential contaminants and removed from the corresponding dataset [36]. Relative abundance plots were generated for the 10 most abundant species across all samples, with values averaged by treatment in both fecal and ruminal samples.

For downstream analysis, samples from the Day 1 group were excluded, and only samples collected on day 70 from the Control and Phytogenic groups were included in the differential abundance analysis. This approach was adopted to evaluate treatment-associated differences after the experimental feeding period, avoiding the influence of baseline microbial composition on the comparison between treatments. Differential taxonomic abundance was assessed using the LEfSe algorithm to identify taxa exhibiting statistically significant differences among groups, thereby elucidating patterns of community assembly [37]. Functional prediction of the microbial communities was performed using Tax4Fun2. Tax4Fun2 predicts functional profiles based on KEGG Orthology (KO) annotations, and the resulting functional profiles were subsequently summarized and differentially represented predicted KEGG pathways between the Control and Phytogenic groups were identified using LEfSe [38]. Alpha diversity of bacterial communities was quantified using Fisher’s alpha index, which is comparatively robust to differences in sample size [39]. Distributions of alpha diversity values across sample groups were visualized using violin plots using Kruskal–Wallis for estimating statistically significant differences. Beta diversity was evaluated by principal coordinate analysis (PCoA) based on Jaccard distance, calculated from presence–absence data, to assess differences in community composition among samples using PERMANOVA [40].

## 3. Results

### 3.1. Zootechnical Performance

The zootechnical performance results are presented in Table 3. The inclusion of the phytogenic blend did not affect body weight, dry matter intake (DMI), or feed efficiency throughout the experimental period. However, animals receiving the phytogenic additive showed greater total weight gain (*p* = 0.05) and higher average daily gain (ADG) (*p* = 0.05) compared with the control group.

### 3.2. Hematological Analysis

The hematological parameters are presented in Table 4. Variables of granulocytes, monocytes, erythrocytes, hemoglobin, hematocrit, and platelets were not affected by treatment. However, a significant treatment × day interaction was observed for total leukocytes (*p* = 0.01) on days 7 and 28, and for lymphocytes (*p* = 0.05) on day 70, with lower values observed in the Phytogenic group.

### 3.3. Serum Biochemistry

Serum biochemical variables are presented in Table 5. Albumin, cholesterol, glucose, uric acid, urea, GGT, and AST did not differ between treatments. However, animals fed with phytogenic additives showed lower creatine kinase (*p* = 0.05) and cholinesterase activities (*p* = 0.04). In addition, total protein (*p* = 0.01) and globulin concentrations (*p* = 0.02) were higher in the Phytogenic group.

### 3.4. LPS, Cytokines, and Proteinogram

Inflammatory and immunological biomarkers are presented in Table 6. The phytogenic group showed lower concentrations of the cytokines as TNF-α and IL-1 (*p* = 0.01), as well as lower levels of C-reactive protein (*p* = 0.05), ferritin (*p* = 0.01), and haptoglobin (*p* = 0.05). In addition, increased concentrations of IL-10 (*p* = 0.01), IgA immunoglobulins (*p* = 0.02), and IgG immunoglobulins (*p* = 0.05) were observed. LPS, ceruloplasmin, and transferrin levels did not differ between treatments.

### 3.5. Oxidative Stress

Oxidative status indicators are presented in Table 7. No significant differences were observed for SOD and PSH. However, TBARS (*p* = 0.02) and ROS (*p* = 0.01) showed a significant treatment × day interaction, with lower values observed in the phytogenic group on days 28 and 70 for TBARS and on day 70 for ROS.

### 3.6. Volatile Fatty Acid Profile in Rumen Fluid and Feces

The results of the VFA profile in rumen fluid and feces are presented in Table 8. Rumen fermentation was influenced by phytogenic intake, with total VFA concentration being higher in the treated group (*p* = 0.01), along with increases in acetate (*p* = 0.01), propionate (*p* = 0.01), butyrate (*p* = 0.01), and isovalerate (*p* = 0.05). Rumen pH was not affected (*p* = 0.92).

**Figure 1:**
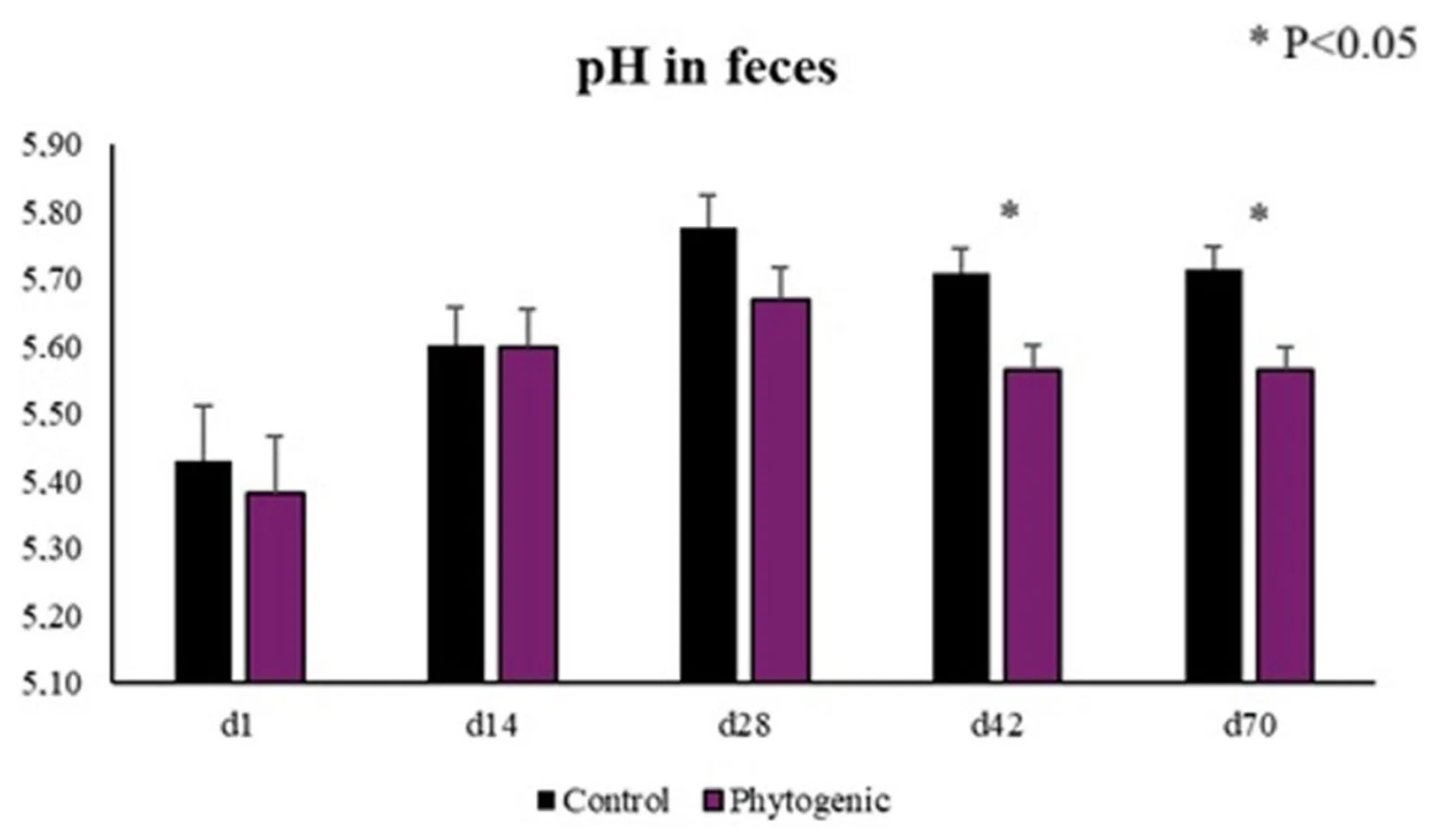
Fecal pH of calves fed a phytogenic additive. Statistical differences (* *p* < 0.05) were observed on days 42 and 70 of the experiment.

Similarly, fecal VFA concentrations (*p* = 0.01) were higher in the treated group, including acetate (*p* = 0.03), propionate (*p* = 0.05), butyrate (*p* = 0.02), and valerate (*p* = 0.05). Fecal pH (Figure 1) showed a time effect, with a significant reduction in the phytogenic group on days 42 and 70 (*p* < 0.05).

### 3.7. Ruminal and Fecal Microbiota Composition and Diversity

**Figure 2:**
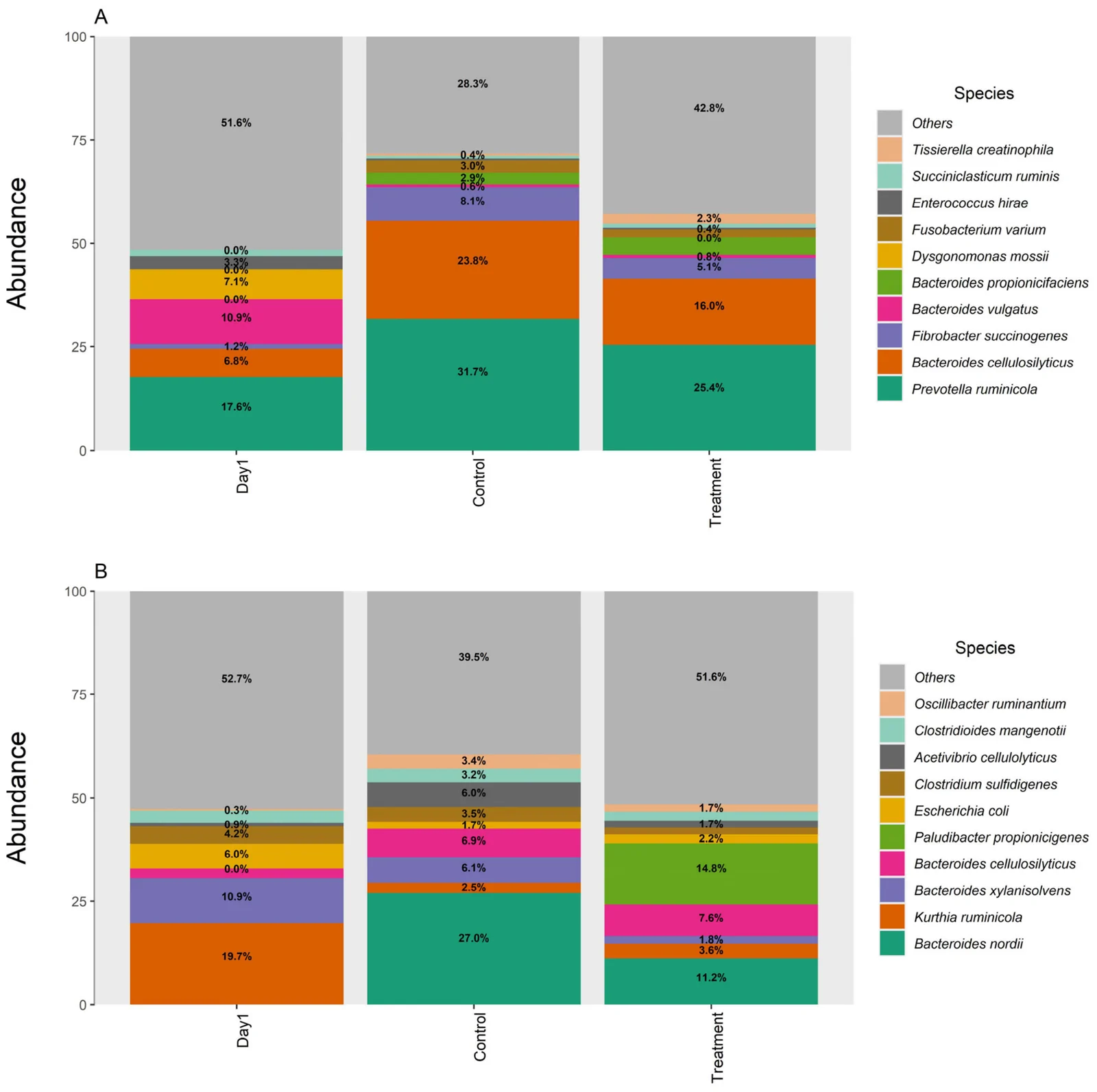
Relative abundance of the predominant bacterial species in ruminal fluid (**A**) and feces (**B**) of calves fed a high-starch diet with or without phytogenic feed. Bars represent the mean relative abundance (%) of the predominant species across samples within each experimental group. The Day 1 group represents the baseline microbiota before the experimental dietary treatment, whereas Control and Treatment represent samples collected after 70 days from calves receiving the high-starch basal diet without or with the phytogenic additive, respectively. The 10 most abundant species were selected for visualization in each biological compartment, and all remaining taxa were grouped as “Others”. Each species is represented by a distinct color, as indicated in the corresponding legend. Relative abundance values are presented as percentages of the total bacterial community.

The relative abundance profiles of the most abundant bacterial species in ruminal fluid and feces are shown in Figure 2. In the ruminal microbiota (Figure 2A), *Prevotella ruminicola* was the predominant species, accounting for 17.6% of the community at day 1, 31.7% in the Control group, and 25.4% in the Treatment group. *Bacteroides cellulolyticus* also increased in relative abundance after 70 days, reaching 23.8% in the Control group and 16% in the Treatment group, compared with 6.8% at day 1. Conversely, *Fibrobacter succinogenes* increased from 1.2% at day 1 to 8.1% and 5.1% in the Control and Treatment groups, respectively.

In the fecal microbiota (Figure 2B), the relative abundance profile differed from that observed in the rumen. The Control group showed a high relative abundance of *Bacteroides nordii* represented 27.0% of the community. The Treatment group showed a higher relative abundance of *Paludibacter propionicigenes* (14.8%), while *Bacteroides xylanisolvens* accounted for 6.1% in the Control group.

**Figure 3:**
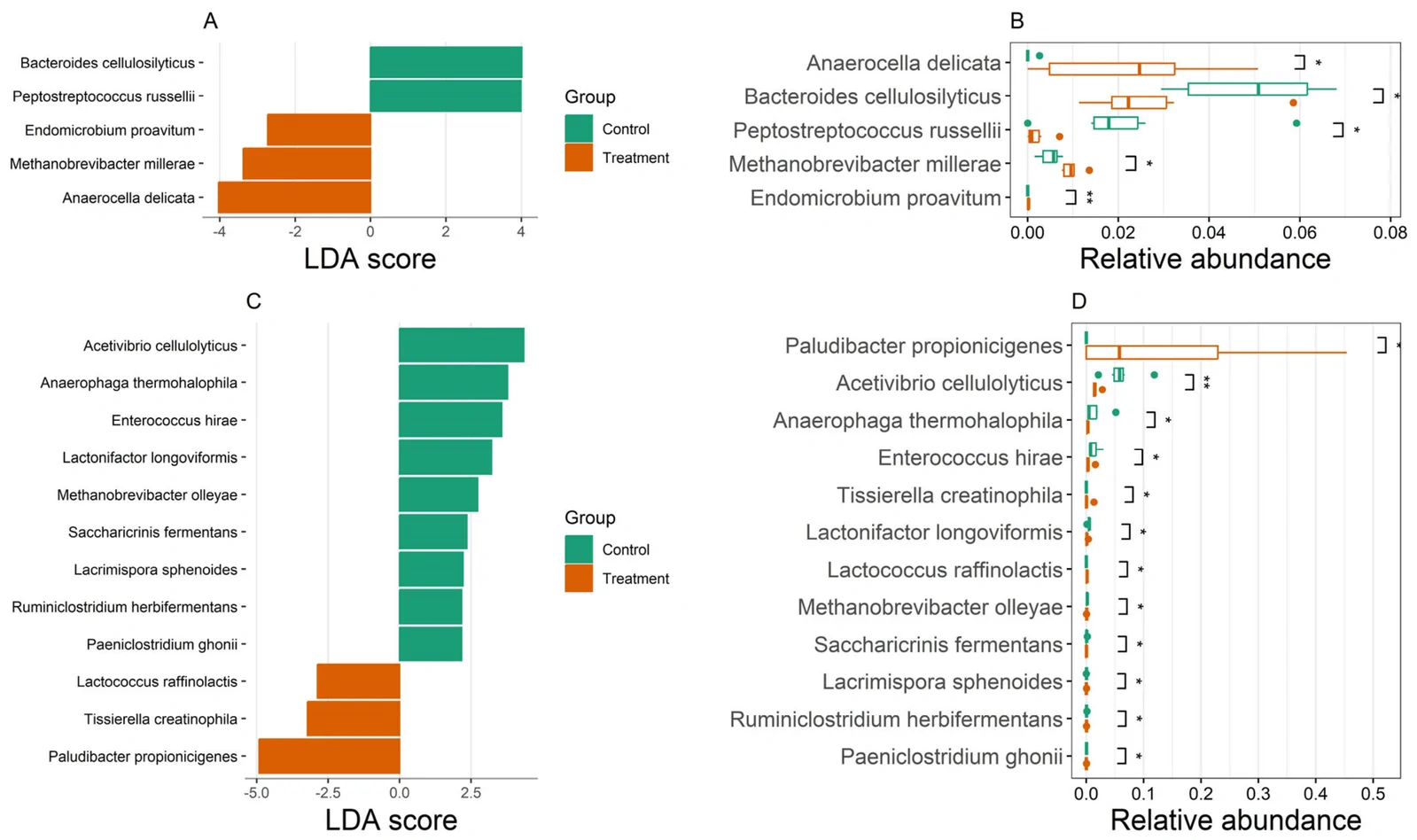
LDA score in ruminal fluid (**A**,**B**) and in feces (**C**,**D**) of bovines fed with phytogenic additive using Green genes2 database. * difference between groups (*p* ≤ 0.05), and ** *p* ≤ 0.01.

Overall, the relative abundance profiles indicate that the phytogenic supplementation was associated with changes in the distribution of the predominant bacterial taxa in both ruminal and fecal communities. However, these relative-abundance profiles are descriptive, and statistical differences among individual taxa were evaluated using LEfSe (Figure 3). Two taxa were enriched in the control group: *Bacteroides cellulosilyticus* (LDA = 4.05; adjusted *p* = 0.037) and *Peptostreptococcus russellii* (LDA = 4.03; adjusted *p* = 0.045). In contrast, three taxa were enriched in the phytogenic group: *Anaerocella delicata* (LDA = 4.05; adjusted *p* = 0.050), *Methanobrevibacter millerae* (LDA = 3.45; adjusted *p* = 0.010), and *Endomicrobium proavitum* (LDA = 2.94; adjusted *p* = 0.007). Thus, the differential analysis indicates that phytogenic supplementation modified specific members of both the bacterial and archaeal ruminal communities rather than producing a uniform shift across all taxa.

In feces, twelve taxa were differentially represented between treatments (Figure 3C,D). Nine taxa were enriched in the control group, including *Acetivibrio cellulolyticus* (LDA = 4.35; adjusted *p* = 0.007), *Anaerophaga thermohalophila* (LDA = 3.83; adjusted *p* = 0.012), *Enterococcus hirae* (LDA = 3.59; adjusted *p* = 0.042), *Lactonifactor longoviformis* (LDA = 3.24; adjusted *p* = 0.012), *Methanobrevibacter olleyae* (LDA = 2.75; adjusted *p* = 0.042), *Saccharicrinis fermentans* (LDA = 2.40; adjusted *p* = 0.041), *Paeniclostridium ghonii* (LDA = 2.23; adjusted *p* = 0.015), *Lacrimispora sphenoides* (LDA = 2.18; adjusted *p* = 0.015), and *Ruminiclostridium herbifermentans* (LDA = 2.14; adjusted *p* = 0.028). Conversely, three taxa were enriched in the phytogenic group: *Paludibacter propionicigenes* (LDA = 4.86; adjusted *p* = 0.025), *Tissierella creatinophila* (LDA = 3.12; adjusted *p* = 0.025), and *Lactococcus raffinolactis* (LDA = 2.86; adjusted *p* = 0.025).

### 3.8. Correlation Between Microbiota and Functional Prediction

**Figure 4:**
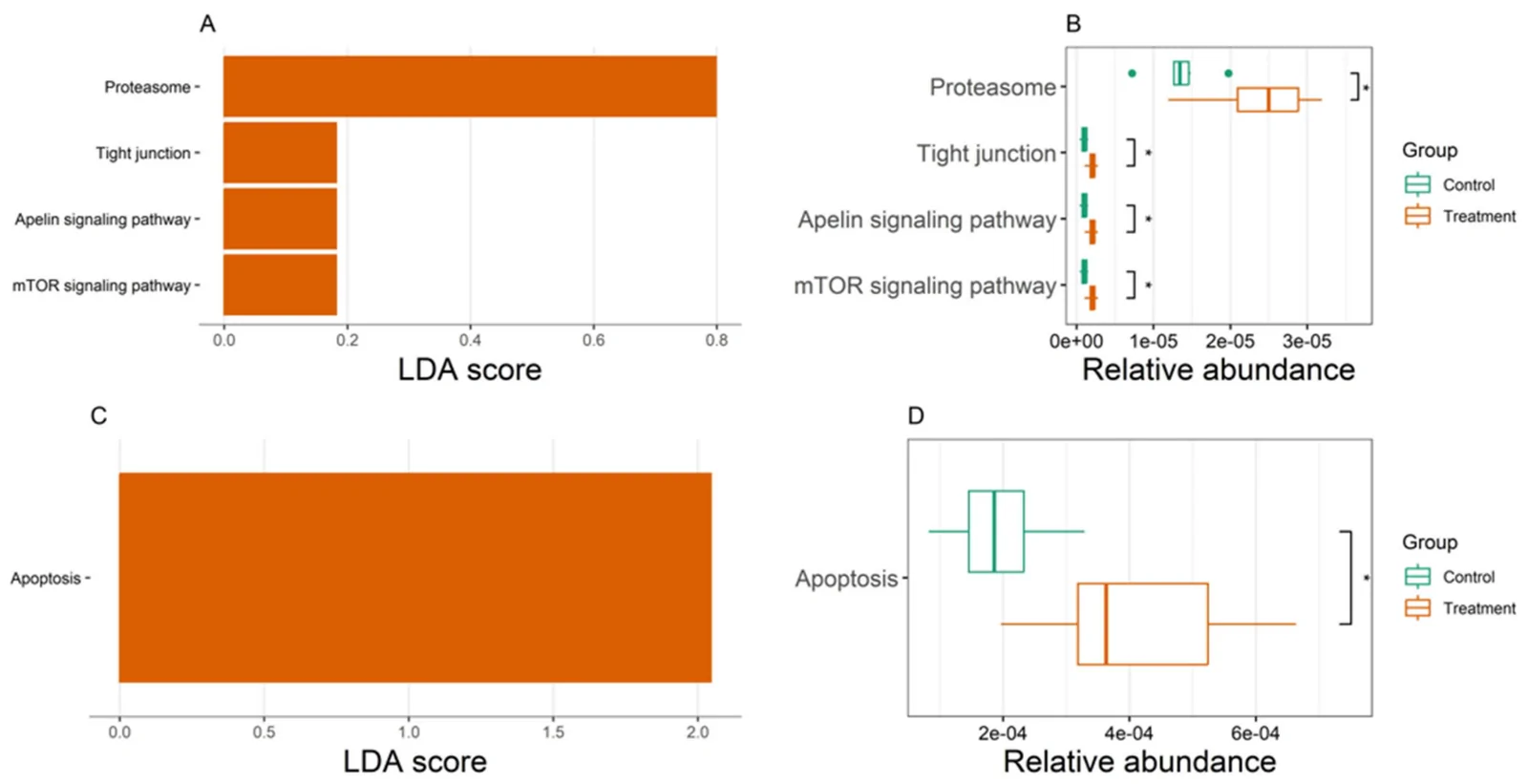
Predicted functional pathways identified by Tax4Fun2 and differential analysis by LEfSe in ruminal fluid ((**A**)—LDA score and (**B**)—Relative abundance of KEGG functions) and feces ((**C**)—LDA score and (**D**)—Relative abundance of KEGG functions) of calves fed phytogenic feed (treatment group) compared to the control group. Statistical differences are represented by brackets with asterisks (* *p* < 0.05).

Tax4Fun2 was used to predict KEGG pathways of the ruminal and fecal microbial communities. (Figure 4). In the rumen, four KEGG pathways were differentially enriched in the phytogenic group (Figure 4A,B). The proteasome pathway showed the highest discriminative capacity, with a greater LDA score than the other pathways and higher relative abundance. In addition, the tight junction, apelin signaling pathway, and mTOR signaling pathway were also significantly enriched in the phytogenic group. In feces, only one KEGG pathway, apoptosis, was differentially represented between treatments (Figure 4C,D), with higher relative abundance in the phytogenic group. Thus, the predicted functional response was broader in the rumen than in the fecal microbiota, indicating that phytogenic supplementation had a stronger effect on the predicted functional potential of the ruminal microbial community.

### 3.9. Microbial Beta and Alpha Diversity

**Figure 5:**
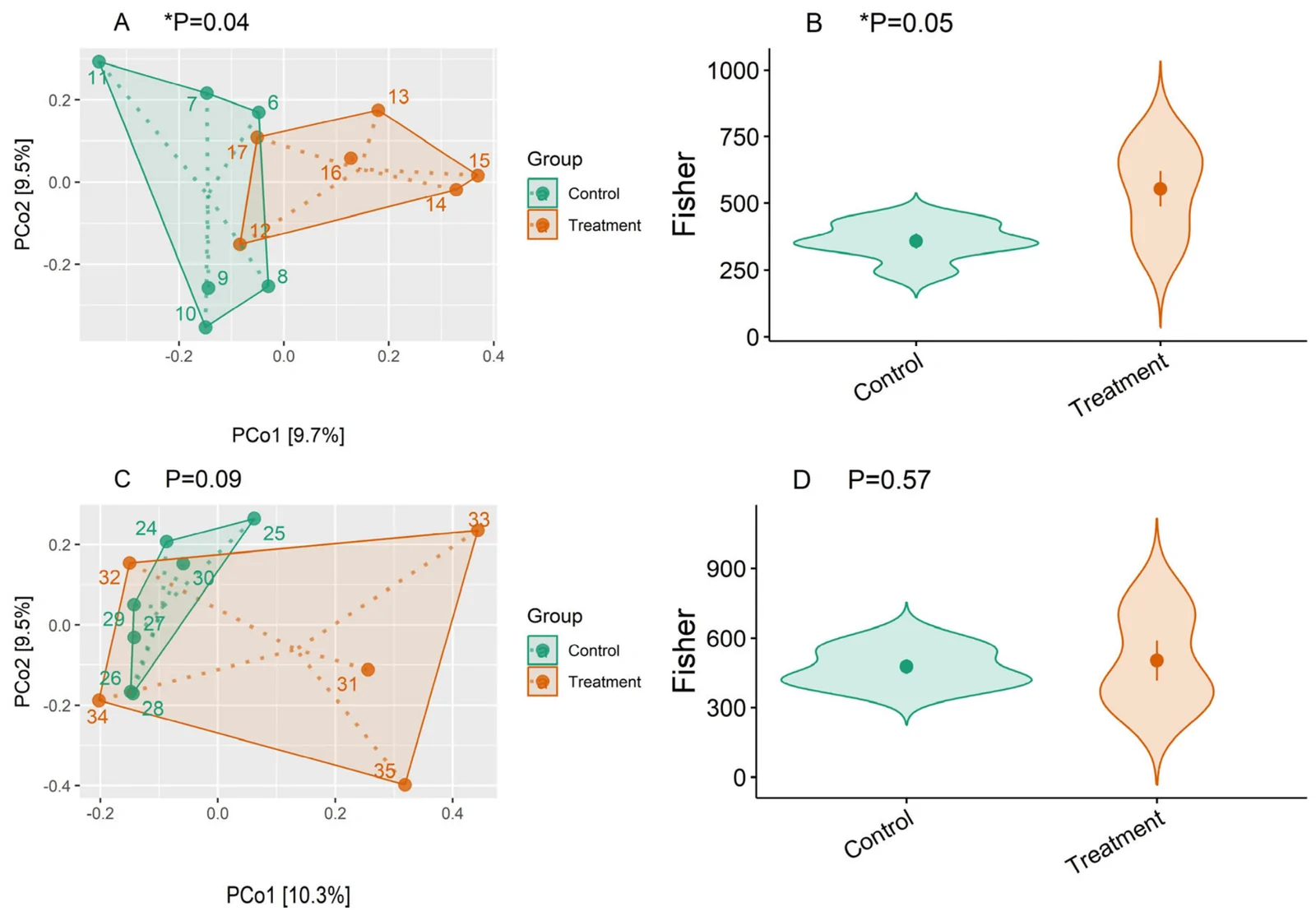
Beta (PCo2) and alpha (Fisher) diversity in rumen fluid (**A**,**B**) and fecal (**C**,**D**) samples from calves fed phytogenic feed. Note that a statistically significant difference was observed between treatments in the rumen (* *p* < 0.05).

Alpha diversity, calculated using the Fisher diversity index, revealed that the active blend altered the internal richness of the environment. Notably, a statistically significant difference in alpha diversity was observed in the ruminal fluid between the treatment and control groups (Figure 5B,C), indicating an increase in microbial richness driven by the additives (*p* < 0.05). However, fecal alpha diversity remained comparable between treatments (Figure 5).

For beta diversity, Principal Coordinate Analysis (PCoA) based on Jaccard distances demonstrated a spatial separation between the ruminal microbiota of control and supplemented calves (Figure 5A,C), confirming that the active blend significantly reshaped the community structure in the rumen (*p* < 0.05). Conversely, fecal beta diversity clusters showed no effect of the treatment in diversity (Figure 5).

## 4. Discussion

The increase in starch in cattle diets has marked effects on their microbiome [5] and influences the expression of inflammatory markers and oxidative status [7], with gastrointestinal dysbiosis and increased inflammation associated with increased risks of gastrointestinal disorders with subclinical ruminal acidosis [41]. The present experiment sought to subject cattle to high levels of dietary starch in a sudden exposure regime, observing effects on the abundance of bacterial phyla in the animals from day 1 to day 70 of the experimental period, appointed for dysbiosis.

Activation of the immune system affects energy expenditure, increasing the demand of glucose for an activated immune system, competing with other biological demands associated with production [42,43]. The use of the blend of actives increased weight gain (Table 3) from day 14 to day 70, with a greater daily measured gain observed in the treated animals, although without differences in dry matter intake and feed efficiency. Animals fed the blend of actives showed a reduction in pro-inflammatory cytokines, acute phase proteins (C-reactive protein, ferritin and haptoglobin), and total leukocytes and lymphocytes, associated with an increase in IL-10, IgA and IgG, globulins, and total proteins, which, accompanied by a reduction in cholinesterase and creatine kinase activity, points to a marked anti-inflammatory response [44], making nutrients available to possibly increase weight gain by reducing demands associated with the immune system. It was observed that ceruloplasmin and transferrin levels were not affected by the treatment; however, the timing of sample collection must be considered, as the levels of these acute-phase proteins might be stabilizing by day 70 of the experiment due to the duration of exposure. The treatment × day interaction observed for total leukocyte and lymphocyte counts indicates that the hematological response to the phytogenic additive varied over time. Lower leukocyte counts in treated animals on days 28 and 70, together with lower lymphocyte counts on day 70, may reflect modulation of systemic immune activity rather than an adverse effect on hematopoiesis. This interpretation is supported by the reduction in pro-inflammatory markers observed in animals receiving the phytogenic blend, together with the increase in IL-10, suggesting a shift toward a more regulated inflammatory response.

Diets rich in starch increase systemic inflammation and elevate oxidative stress, being associated with increased activity of the transcription factor NF-kB and aerobic glycolysis [7,45]. The use of the active blend reduced serum ROS and TBARS levels, demonstrating a reduction in oxidants and lipid peroxidation. This effect is supported by the reduction in systemic inflammation and the antioxidant activity of the essential oils that make up the blend of actives, such as silymarin, eugenol, carvacrol, and cinnamaldehyde [19,46]. Exposure to diets with high levels of concentrate increases portal uptake of LPS, with the blend having no effect on this mechanism; this suggests local alterations in the capacity to maintain barrier integrity or in the transcellular absorption of LPS [17], although the specific mechanism was not investigated in this study. However, it was observed that the treatment reduced levels of inflammatory markers despite LPS levels, pointing to anti-inflammatory regulation that mitigates the effects of serum LPS.

Maintaining a stable rumen environment is crucial for the balance of the rumen microbiota and for maximum performance [2]. Volatile fatty acids from rumen fermentation are responsible for 70% of the ruminant’s energy intake and are also associated with increased microbial protein [47]. The addition of yeasts and their metabolites, such as beta-glucans and mannan oligosaccharides, helps stabilize the rumen’s redox potential and modulate the immune system and gastrointestinal microbiota, resulting in positive effects on health and performance [10,15]. The blend of actives does not affect rumen pH but increased volatile fatty acid levels with an increase in acetic, propionic, butyric, and isovaleric acids (Table 8) with effects on the ruminal microbiota (Figure 2 and Figure 3) reducing dysbiosis and supporting greater weight gain (Table 3). The increase in fecal VFA concentrations was accompanied by a reduction in fecal pH in the phytogenic group on days 42 and 70 (Figure 1), suggesting enhanced hindgut fermentation in supplemented animals.

High-starch diets can substantially alter ruminal and hindgut microbial communities by modifying substrate availability and fermentation [48]. The enrichment of *M. millerae* is of particular interest because this archaeon is a recognized hydrogenotrophic methanogen of the bovine rumen [49]. In feces, the enrichment of *P. propionicigenes*, a propionate-producing anaerobe [50], was compatible with the higher fecal VFA concentrations observed in supplemented animals.

The microbiota alpha and beta diversity analyses showed significant differences between treatments in the rumen, whereas neither alpha nor beta diversity differed significantly in feces. This indicates that the phytogenic blend produced a broader restructuring of the ruminal community, while the fecal response was more selective. These findings are consistent with the concept that high-starch diets can substantially reshape bovine ruminal and hindgut microbiota [51], while suggesting that the phytogenic blend modulated this microbial response and altered fermentation without eliminating the fundamental microbial adaptations induced by the high-starch diet.

## 5. Conclusions

This experiment demonstrated the effect of the active ingredient blend in animals subjected to high-starch diets. The use of the active ingredient blend reduced the inflammatory response associated with increased dietary starch, with a decrease in acute phase proteins, total leukocytes, lymphocytes, inflammatory cytokines, serum cholinesterase and creatine kinase activity, and a reduction in lipid peroxidation and ROS levels. Animals that received the blend showed higher LDA and beta diversity of the ruminal microbiota, associated with increased levels of total volatile fatty acids, indicating a protective effect of the blend against dysbiosis induced by the high-starch diet. Together, these findings suggest that the phytogenic blend modulated ruminal microbial ecology and gastrointestinal fermentation and may contribute to improved physiological adaptation to high-starch feeding.

## Figures and Tables

**Table 1:** Chemical and microbiological composition of the experimental additive LactoPremium^®^ per 100 g of product.

Active Ingredient	Source Product	Concentration per 100 g
Sanguinarine	Sangrovit^®^	50.00 mg *
Chelerythrine	Sangrovit^®^	16.67 mg
Carvacrol	Activo-O.E^®^	600.00 mg
Cinnamaldehyde	Activo-O.E^®^	450.00 mg
Eugenol	Activo-O.E^®^	150.00 mg
Capsaicin	Activo-O.E^®^	50.00 mg
*Saccharomyces cerevisiae*	Procreatin^®^	9.0 × 10^10^ CFU
Polycationic Bentonite	Yes Fix HP^®^	20.00 g
Silymarin	Yes Fix HP^®^	8.00 g
β-glucans	Yes Fix HP^®^	2.00 g
Organic Selenium	Yes Fix HP^®^	40.00 mg

* Milligrams = mg; Grams = g; Colony Forming Units = CFU.

**Table 2:** Chemical composition of the basal diet fed to the cattle.

Variables	TMR (% of DM)
Dry matter (DM)	51.5
Crude protein (CP)	14.1
Ether Extract (EE)	2.98
Neutral detergent fiber (NDF)	35.2
Starch	33.9
Total Digestible Nutrients (TDN)	71.5

**Table 3:** Zootechnical performance of male Holstein cattle who consumed a commercial product developed by combining other commercial phytogenic-based products.

Variables	Control	Phytogenic	SEM	*p*-Treat ^1^	*p*: Treat × Day ^2^
Body weight, kg				0.55	0.23
d1	181	181	2.25		
d14	200	200	2.21		
d28	225	227	2.29		
d70	260	266	2.14		
Weight gain (d14–70), kg	60.5 b	65.7 a	1.75	0.05	NE
ADG, kg	1.08 b	1.17 a	0.03	0.05	NE
Dry matter intake (DMI)	4.73	4.76	0.21	0.96	0.97
Feed efficiency, kg/kg	0.228	0.245	0.02	0.12	NE

Note ^1^. Treatment effect, when *p* ≤ 0.05, indicated by different letters within the same row. Note ^2^. Treatment × day interaction, when *p* ≤ 0.05, indicated by letters within the same row. NE—not evaluated.

**Table 4:** Hemogram of male Holstein cattle who consumed a commercial product developed by combining other commercial phytogenic-based products.

Variables	Control	Phytogenic	SEM	*p*: Treat ^1^	*p*: Treat × Day ^2^
Total Leukocytes (×10^3^ µL)	8.12	7.18	0.37	0.21	0.01
d1	8.54	8.39	0.36		
d14	6.93	6.97	0.36		
d28	8.31 a	6.73 b	0.35		
d70	9.13 a	7.85 b	0.35		
Lymphocyte (×10^3^ µL)	4.39	4.01	0.2	0.46	0.05
d1	4.66	4.51	0.21		
d14	3.81	4.21	0.22		
d28	4.72	3.93	0.19		
d70	4.79 a	3.82 b	0.19		
Granulocytes (×10^3^ µL)	2.65	2.16	0.19	0.75	0.54
Monocytes (×10^3^ µL)	1.07	1	0.05	0.92	0.95
Erythrocytes (×10^6^ µL)	7.34	6.91	0.25	0.89	0.91
Hemoglobin (mg/dL)	9.75	9.47	0.33	0.92	0.94
Hematocrit (%)	28.4	27.6	1.01	0.9	0.94
Platelets (×10^3^ µL)	285	276	15.1	0.88	0.72

Note ^1^. Treatment effect, when *p* ≤ 0.05, indicated by different letters within the same row. Note ^2^. Treatment × day interaction, when *p* ≤ 0.05, indicated by letters within the same row.

**Table 5:** Serum biochemistry of male Holstein cattle who consumed a commercial product developed by combining other commercial phytogenic-based products.

Variables	Control	Phytogenic	SEM	*p*: Treat ^1^	*p*: Treat × Day ^2^
Albumin (g/dL)	2.99	2.89	0.03	0.75	0.82
Creatine Kinase (U/L)	384 a	351 b	10.2	0.05	0.24
Cholinesterase (U/L)	228 a	210 b	5.76	0.04	0.09
Cholesterol (mg/dL)	70.9	63.4	3.27	0.66	0.43
Glucose (mg/dL)	86.4	93.3	1.99	0.15	0.08
Uric acid (mg/dL)	0.66	0.63	0.03	0.91	0.96
Urea (mg/dL)	13.7	13.8	0.57	0.95	0.97
Total protein (g/dL)	5.88 b	6.97 a	0.08	0.01	0.07
Globulin (g/dL)	2.89 b	4.08 a	0.09	0.02	0.11
GGT (U/L)	15.9	15.5	0.51	0.96	0.91
AST (U/L)	87.3	85.1	3.03	0.94	0.86

Note ^1^. Treatment effect, when *p* ≤ 0.05, indicated by different letters within the same row. Note ^2^. Treatment × day interaction, when *p* ≤ 0.05, indicated by letters within the same row.

**Table 6:** LPS, cytokines, and protein profile of male Holstein cattle on day 70 of the experiment.

Variables	Control	Phytogenic	SEM	*p*: Treat ^1^
LPS (EU/mL)	37.1	38.4	1.25	0.90
TNF-α (pg/mL)	254 a	197 b	7.47	0.01
IL-1 (pg/mL)	74.2 a	62.1 b	2.51	0.01
IL-10 (pg/mL)	102 b	135 a	4.21	0.01
C-reactive protein (g/dL)	0.154 a	0.123 b	0.005	0.05
Ferritin (g/dL)	0.259 a	0.187 b	0.009	0.01
Ceruloplasmin (g/dL)	0.651	0.614	0.09	0.46
Haptoglobin (g/dL)	0.354 a	0.302 b	0.03	0.05
Transferrin (g/dL)	0.379	0.406	0.09	0.16
IgA (mg/dL)	05.63 b	08.95 a	0.06	0.02
IgG (mg/dL)	10.61 b	13.87 a	0.053	0.05

Note ^1^. Treatment effect, when *p* ≤ 0.05, indicated by different letters within the same row.

**Table 7:** Oxidative status of male Holstein cattle who consumed a commercial product developed by combining other commercial phytogenic-based products.

Variables	Control	Phytogenic	SEM	*p*: Treat ^1^	*p*: Treat × Day ^2^
SOD (U/mg protein)	2.33	2.39	0.03	0.92	0.86
PSH (µmol/L)	6.51	6.62	0.15	0.87	0.91
TBARS (nmol/mL)	10.7	9.61	0.23	0.68	0.02
d1	10.7	11.2	0.27		
d14	10.4	10.8	0.27		
d28	10.7 A	9.59 B	0.22		
d70	10.9 A	8.41 B	0.17		
ROS (U fluorescence)	7.76	7.09	0.45	0.59	0.01
d1	11.3	11.6	0.44		
d14	8.45	8.36	0.47		
d28	7.91	8.13	0.42		
d70	6.92 A	4.79 B	0.43		

Note ^1^. Treatment effect, when *p* ≤ 0.05, indicated by different letters within the same row. Note ^2^. Treatment × day interaction, when *p* ≤ 0.05, indicated by letters within the same row.

**Table 8:** Profile of volatile fatty acids (VFA) in the rumen and feces of male Holstein cattle on day 70 of the experiment.

Variables	Control	Phytogenic	SEM	*p*: Treat ^1^
Ruminal liquid				
Total VFA (mmol L^−1^)	32.2 b	50.75 a	2.41	0.01
Acetic acid (mmol L^−1^)	23.3 b	35.0 a	1.74	0.01
Propionic acid (mmol L^−1^)	5.75 b	9.96 a	0.57	0.01
Isobutyric acid (mmol L^−1^)	0.42	0.72	0.19	0.09
Butyric acid (mmol L^−1^)	1.90 b	3.40 a	0.32	0.01
Isovaleric acid (mmol L^−1^)	0.54 b	1.17 a	0.11	0.05
Valeric acid (mmol L^−1^)	0.25	0.41	0.09	0.14
pH	6.64	6.71	0.02	0.92
Feces				
Total VFA (mmol L−1)	51.6 b	64.8 a	2.98	0.01
Acetic acid (mmol L^−1^)	36.2 b	47.1 a	1.35	0.03
Propionic acid (mmol L^−1^)	8.81	10.63	0.58	0.05
Isobutyric acid (mmol L^−1^)	0.28	0.28	0.04	0.96
Butyric acid (mmol L^−1^)	6.12 b	8.41 a	0.38	0.02
Isovaleric acid (mmol L^−1^)	0.14	0.13	0.02	0.98
Valeric acid (mmol L^−1^)	0.07 b	0.25 a	0.07	0.05

Note ^1^. Treatment effect, when *p* ≤ 0.05, indicated by different letters within the same row.

## Data Availability

The data presented in this study are available from the corresponding author upon request. The restriction is due to privacy concerns, as this dataset is part of a larger study and will be used in future publications.

## Supplementary Materials

The following supporting information can be downloaded at: https://www.mdpi.com/article/10.3390/ani16172803/s1, Table S1: Standardization of short-chain fatty acid analysis in feces and ruminal fluid.
