# Supplementary material for: The Combination of Phytogenic in High-Starch Diets for Growing Cattle and Its Effects on Immunomodulation, Fermentation, Microbiota, and Performance
Source: Animals (Basel). 2026 Sep 6;16(17):2803. doi: 10.3390/ani16172803 (PMC13565058; doi:10.3390/ani16172803)
Supplement: Supplementary file 1 [file animals-16-02803-s001.zip › animals-4438312-supplementary.pdf]

Table S1. Standardization of short-chain fatty acid analysis in feces and ruminal fluid.

Item	**Acetic acid**	**Propionic acid**	**Butyric acid**	**Isovaleric acid**
R^2	0.9998	0.9998	0.9998	0.9997
Equation	y = 0.0191x + 0.0089	y = 0.0328x + 0.01	y = 0.0488x + 0.0024	y = 0.0652x + 0.0016
Linear range (mmol kg^{-1})*	1.01 - 24.16	0.43 - 20.75	0.17 - 10.76	0.13 - 4.03
LOD (mmol kg^{-1})	0.06	0.05	0.04	0.02
LOQ (mmol kg^{-1})	0.13	0.11	0.08	0.03
Accuracy (%)	103.47	90.21	101.50	102.54
Repeatability (RSD)	2.93	4.06	4.46	4.32

* The linear range, LOD (limit of detection) and LOQ (limit os quantitation) were expressed in mmol of SFA for kg of feces.
